# Evaluation of non-genetic factors affecting calf growth, reproductive performance and milk yield of traditionally managed Sheko cattle in southwest Ethiopia

**DOI:** 10.1186/s40064-015-1340-9

**Published:** 2015-10-01

**Authors:** E. Bayou, A. Haile, S. Gizaw, Y. Mekasha

**Affiliations:** Haramaya University, P.O. Box 138, Dire Dawa, Ethiopia; International Centre for Agricultural Research in the Dry Areas, P.O. Box 5689, C/O ILRI Addis, Addis Ababa, Ethiopia; International Livestock Research Institute (ILRI), P.O. Box 5689, Addis Ababa, Ethiopia

**Keywords:** Lowland, Midland, Monitoring, Non-genetic, Performance, Sheko

## Abstract

The study was conducted to estimate calf growth, reproductive performance and milk yield of Ethiopia Sheko cattle and to assess non-genetic factors affecting their performance in their home tract as a step towards designing sustainable cattle conservation and improvement strategy. All the growth traits considered in the study were significantly affected by all non-genetic factors considered except for the fixed effects of Agro ecological zones (AEZs) and season of birth which were not significant for post weaning daily gain. Calving interval (CI) and days open (DO) were significantly influenced by AEZs, season and dam parity. Cows that calved in lowland had shorter CI and DO than cows which calved in midland. Cows that calved in short rainy season had Short CI and DO than those calved during dry season or long rainy season. Cows which calved for the first time had the longest CI and DO from the other parities whereas cows on their fifth parity had the shortest CI and DO. AEZ significantly affected lactation milk yield (LMY) and lactation length (LL), but not significant on daily milk yield (DMY) and 305 days yield (305DY). Season was significant on all milk traits considered except DMY. Parity effect was significant on LMY and 305DY, whereas DMY and LL were not affected. The non-genetic factors had significant effects for all of the reproductive; and many of the growth and milk performance traits considered and hence will need to be considered in cattle breed improvement program.

## Background

Although, cattle have a very important role in the economies of the farmers and the country at large, population of some of the valuable breeds is either declined or extinct (Dadi et al. 2008). According to Rege (1999), two Ethiopian indigenous cattle breeds (Arsi-Sanga and Kuri) are already reported as extinct while the Sheko breed is endangered of extinction. Sheko cattle are the last remnants of the original Hump less shorthorn (*Bos taurus*) cattle in East Africa. This breed is among the cattle breeds of Ethiopia that have been traditionally kept by small number of local farmers in the humid Sheko and Bench districts under mixed crop-livestock farming systems of Southwest Ethiopia. The breed is known for their better performance and ability to survive, produce and reproduce in tsetse infested areas. The breed is believed to have some level of trypanotolerance (DAGRIS 2004). Today, the majority of Sheko cattle manifest small humps that they inherited from zebu cattle. Sheko is now considered endangered by gradual interbreeding with local zebu (DAGRIS 2004). Despite the fact that the superior performance of Sheko breed is widely recognized by Sheko keepers, local authorities and also by Sheko breeders outside the area, Sheko cattle face a number of different threats to their survival. This calls for urgent breed conservation and improvement program.

Detailed and up-to-date information on performance parameters of growth, reproduction and milk yield of the breed and non-genetic factors affecting their performances need to be considered for sustainable breed improvement and conservation program (Negussie et al. 1998). Unfortunately, this information is not readily available for the breed and available information so far has been based on the continuous recalling method interviews of rapid surveys which do not reflect the real performance of the breed although they can be used as baseline.

Thorough monitoring of growth, reproductive and productive performance of the breed in their habitat under farmer’s condition is required to capture a full picture of their contribution. Therefore, the objectives of this study were to estimate calf growth, reproductive performance and milk yield of Sheko cattle breed and evaluate non-genetic factors affecting their performance under smallholder farmer’s management condition to facilitate breed conservation and improvement program.

## Methods

### The study area and sampling framework

Before starting the monitoring, a preliminary survey was conducted before the main survey to identify the distribution and major areas of concentration of Sheko cattle breed in the study area. Based on the reconnaissance survey and secondary information gathered from the district office of agriculture, three districts, namely Sheko, Semein Bench and Debub Bench of Bench Maji Zone, south western Ethiopia, that are known for keeping Sheko cattle were identified. The districts were further classified, based on agro-ecology into lowland and midland agro ecological set ups. This was followed by identification of four peasant associations (PAs) (2 from midland and 2 from lowland) from each district. These included Mehal Sheko, Shayita, Boyita and Fajek from Sheko district, Genja, Endekael, Wushikin and Garikin from Semein Bench, and Miah, Kokin, Kitte and Zemika from Debub Bench, where the first 2 PAs represent midland while the other 2 represent lowland. The PAs were selected purposively based on concentration of Sheko cattle, their suitability for cattle production, accessibility to market and road, availability of common grazing land and willingness of the farmers to participate in the program. Sampling of households was done by setting criteria; having at least one pregnant Sheko cow or heifer and willingness to participate in the monitoring study. A total of 75 pregnant Sheko cows and/or heifers owned by 75 farmers (43 from lowland and 32 from midland) were included in the monitoring study. In monitoring study, the majority of the data were collected by the research scientists, and not in a participatory manner (less reliable than observation). BMZARDO (2012) briefly described the study areas below.

Sheko district lies between a latitude and longitude of 6°50′N and 35°00′E coordinates, respectively, and at an altitude that ranges from 950 to 1800 m above sea level (m.a.s.l.). The major area is classified under warm and humid to per-humid. The mean annual temperatures were 22.6 °C and the annual rainfall at Mehal Sheko town (the capital of Sheko district) ranges from 1200 to 2200 mm per year. The soil type includes red brown and sandy loam.

Semein Bench district is classified under humid to per-humid climatic condition. It lies at an altitude ranging from 1050 to 2400 m.a.s.l. Average annual temperature ranges from 21.3 to 26 °C and the mean annual rainfall of the district varied from 1200 to 2200 mm per annum. The soil type includes red brown, deep reddish, sandy loam and clay.

Debub Bench district is classified under warm and humid zone to per-humid climatic condition. Its altitude ranges from 980 to 1900 meters above sea level. The average total annual rainfall is 1800 mm and the mean annual maximum and minimum temperatures recorded in were 17.25 and 27.5 °C. The predominant soil types of the area include red brown, deep reddish and sandy loam.

### Data collection and analysis

Each experimental animal was identified such that the animal records to give complete information on calf sex, site, parity, calving date, calf birth weight and calf weight at different ages, daily milk yield, date of drying off and the next successful mating or conception date and calving date. The traits studied for growth performance included birth weight (BW), 6-months weight (6 MW), weaning weight (WW) at 9 month, yearling weight (YW), 18-months weight (18 MW), pre weaning daily body weight gain (birth to weaning age) (PrWDG) and post weaning daily body weight gain (weaning to eighteen age) (PoWDG). Calf birth weight and calf weight at different ages was estimated using heart girth-weight conversion tape developed for the breed (OARI 2002). Average pre weaning daily weight gain (PrWDG) and post weaning daily weight gain (PoWDG) were computed as ADG_t2–t1_ = (W_t2_–W_t1_)/t2–t1 where ADG_t2-t1_ is the weight gain between periods t1 and t2, W_t2_ the weight at age t2, W_t1_ the weight at age t1 and t2–t1 is the number of days between ages t1 and t2. The milk yield of the selected animals was measured using plastic measuring cylinder and registered on the prepared format twice a month, morning and evening, for a single day milk yield until the end of lactation to estimate average daily milk yield (DMY), lactation milk yield (LMY), 305 days yield (305DY), and lactation length (LL). DMY in 24 h were calculated by summing each of the test days (morning and evening) milk yields and dividing it by the number of test days (morning and evening). While multiplying average lactation length and daily milk yield give total lactation milk yield. In the study of total lactation milk yield, complete lactation test day records of daily milk yields from calving to end of lactation (lactation length) were included. Therefore, from each cow file containing complete lactation records, a total number of 1522 lactation records were taken. The data consisted of total lactation milk yields of vary lactation lengths. Some cows had less than 305 day’s lactation length. Animals that fail to show estrus sign while lactating remain in lactation, resulting in lactation length well over 305 days, even more than a year. The 305-day milk yield was defined as milk yield up to 305-days of lactation. In this study for all test day lactation record below and over 305 days in lactation were not used in calculation and only the test day milk recorded up to 305 day location were summed up and taken as the actual 305 days yield. For 305-day milk yield study, the final data file contained 547 milk records on test days of Sheko cows. Days open (DO) was calculated from date of last parturition to the next successful mating or conception date and calving interval (CI) was calculated by adding gestation length and days open. Growth performance (2002–2004) data of 75 calves, reproductive performance (2002–2004) data of 75 cows and milk yield data on 1522 milk records on test days (2002–2004) of 75 cows were made available for analysis. Analysis of variance of fixed effects was done by using General Linear Model (GLM) procedures of the Statistical Analysis System (SAS, release 9.1, 2004). The fixed effects of sex of calf’s (male and female), agro ecological zones (lowland agro ecological zone [from 500 to 1500 masl] and midland agro ecological zone [above from 1500 to 2300 masl]), season (main rainy season [June–October], dry season [November–February] and short rainy season [March–May]) and dam parity (1, 2, 3, 4 and 5) on growth traits were investigated. All factors except sex of calf’s were used in investigating the different reproductive and milk performances traits of cows. Stage of lactation was specific character of DMY only so that analysis of stage of lactation (early lactation [1–3 months], mid lactation [4–6 months] and late lactation [≥7 months]) was employed for DMY only by fitting lactation stage as fixed factor. Probability of differences was used to determine any significance differences between the least squares means.

Model used to analyze calf weight was:

$$ {\text{Y}}_{ijklm} = \mu + {\text{S}}i + {\text{A}}j + Se_{k} + {\text{P}}_{l} + {\text{ e}}_{\text{ijklm}} $$

where, Y_*ijklm*_ is the observation on birth weight, weight gains and weight at different ages, µ is the overall mean, S*i* is the fixed effect of *i*th sex of the calf, A*j* is the fixed effect of *j*th agro ecology, *Se*_*k*_ is the fixed effect of *k*th season of calving of the cow, P_*l*_ is the fixed effect of *l*th dam parity, e_*ijklm*_ is the effect of random error

Model used to analyze reproductive performance was:

$$ {\text{Y}}_{ijkl} = \mu + {\text{A}}_{i} + S_{j} + {\text{P}}_{k} + {\text{e}}_{ijkl} , $$

where, Y_*ijkl*_ is the observation on CI and DO, µ is the overall mean, A_*i*_ is the fixed effect of *i*th agro ecology, *S*_*j*_ is the fixed effect of *j*th season of calving of the cow, P_*k*_ is the fixed effect of *k*th dam parity, e_*ijkl*_ is the effect of random error.

Model used to analyze milk performance was:

Model used for DMY was:

$$ {\text{Y}}_{ijkl} = \mu + {\text{A}}_{i} + S_{j} + {\text{P}}_{k} + {\text{L}}_{\text{l}} + {\text{e}}_{ijklm} , $$

where, Y_*ijkl*_ is the observation on DMY, µ is the overall mean, A_*i*_ is the fixed effect of *i*th agro ecology, *S*_*j*_ = fixed effect of *j*th season of calving of the cow, P_*k*_ is the fixed effect of *k*th dam parity, L_l_ is the fixed effect of *l*th stage of lactation, e_*ijklm*_ is the effect of random error

Model used for LMY, 305YD and LL was

$$ {\text{Y}}_{ijkl} = \mu + {\text{A}}_{i} + S_{j} + {\text{P}}_{k} + {\text{e}}_{ijkl} , $$

where, Y_*ijkl*_ is the observation on LMY, 305YD and LL, µ is the overall mean, A_*i*_ is the fixed effect of *i*th agro ecology, *S*_*j*_ is the fixed effect of *j*th season of calving of the cow, P_*k*_ is the fixed effect of *k*th dam parity, e_*ijkl*_ is the effect of random error

## Results and discussion

### Effects of non-genetic factors

#### Growth performance

The overall least squares means and standard errors observed for BW, 6 MW, WW, YW and 18 MW were 16.12 ± 0.22, 58.84 ± 0.51, 76.29 ± 0.45, 85.07 ± 0.50 and 111.62 ± 0.74 kg, respectively and the values for PrWDG and PoWDG were 222.02 ± 1.56 and 129.44 ± 1.78 gm/day, respectively. BW obtained in this study for Sheko calves were lower than values reported by other studies for Boran, Horro and Barka breed (Demeke et al. 2003; Aynalem 2006), Fogera breed (Addisu et al. 2010) and Ogaden calves (Getinet et al. 2009).

In general these estimates are lower than the on-station growth performance of the Horro cattle breed reported in the literature (Demeke et al. 2003; Jirenga et al. 2006; Habtamu et al. 2008). The finding was also lower than the on-station growth performance reported for indigenous Fogera breed (Giday 2001; Amsalu 2003; Addisu et al. 2010) and Ogaden breed (Getinet et al. 2009) but higher than the value from the findings of Mekonen et al. (2011), obtained in monitoring study of Horro cattle. Growth performance of animal in the face of multiple stresses (nutrition, heat, parasites, disease and poor management), when small holder farmers are most vulnerable are the most important factors, resulting lower growth performance. Therefore manipulation of the management system could easily result in improved growth performance. Report by Kosgey (2004) indicated that improvement in performance can be achieved through improvement in management and feeding conditions, and through genetic improvement by use of selecting genetically superior animals. Least squares means and standard errors for effects of non genetic factors on growth performance traits are presented in Table 1.

**Table 1:** Least squares means (±SE) for effects of non genetic factors on growth parameters

Effects/levels	N	BW (kg)	6 MW (kg)	WW (kg)	YW (kg)	18 MW (kg)	PrWDG (gram)	PoWDG (gram)
Overall	75	16.12 ± 0.22	58.84 ± 0.51	76.29 ± 0.45	85.07 ± 0.50	111.62 ± 0.74	222.02 ± 1.56	129.44 ± 1.78
R^2^		0.84	0.79	0.74	0.75	0.71	0.67	0.31
CV %		5.73	3.90	3.02	2.90	3.55	3.96	11.30
Sex		**	**	**	**	**	**	**
Male	39	17.25 ± 0.15	59.33 ± 0.39	77.54 ± 0.39	87.18 ± 0.42	113.62 ± 0.68	222.34 ± 1.50	132.18 ± 2.49
Female	36	15.83 ± 0.15	54.59 ± 0.41	73.06 ± 0.41	82. 82 ± 0.44	106.13 ± 0.71	210.76 ± 1.58	120.55 ± 2.64
AEZ		**	**	**	**	**	**	NS
Low	43	17.15 ± 0.15	58.87 ± 0.40	77.63 ± 0.40	88.18 ± 0.43	112.80 ± 0.69	223.05 ± 1.52	128.78 ± 2.53
Mid	32	15.92 ± 0.16	55.05 ± 0.42	72.97 ± 0.42	81.82 ± 0.45	106.95 ± 0.73	210.05 ± 1.62	123.95 ± 2.69
Season		**	**	**	**	**	**	NS
Main	24	16.33 ± 0.20^a^	56.66 ± 0.52^a^	73.70 ± 0.52^a^	87.20 ± 0.56^b^	107.66 ± 0.90^a^	211.43 ± 2.0^a^	124.69 ± 3.33^a^
Dry	20	18.14 ± 0.25^b^	54.46 ± 0.67^b^	77.20 ± 0.37^b^	83.51 ± 0.72^a^	109.74 ± 1.15^a^	208.99 ± 2.55^a^	126.3 ± 4.25^a^
Short	31	15.14 ± 0.13^c^	59.76 ± 0.34^c^	74.99 ± 0.67^a^	84.29 ± 0.36^a^	112.23 ± 0.58^b^	229.22 ± 1.29^b^	128.11 ± 2.14^a^
Parity		**	**	**	**	**	*	**
1st	22	14.82 ± 0.20^a^	54.61 ± 0.50^a^	73.02 ± 0.50^a^	82.58 ± 0.54^a^	106.51 ± 0.87^a^	214.48 ± 1.92^a^	123.33 ± 3.19^a^
2nd	14	16.29 ± 0.24^b^	56.19 ± 0.61^b^	75.17 ± 0.61^b^	85.05 ± 0.66^b^	109.58 ± 1.05^b^	216.31 ± 2.34^a^	125.81 ± 3.89^a^
3rd	12	18.04 ± 0.18^c^	60.22 ± 0.45^c^	76.59 ± 0.46^c^	86.00 ± 0.49^b^	113.95 ± 0.79^c^	215.06 ± 1.74^a^	136.97 ± 2.90^b^
4th	17	16.48 ± 0.33^b^	57.06 ± 0.82^b^	77.47 ± 0.82^c^	86.55 ± 0.88^b^	111.43 ± 1.42^bc^	224.94 ± 3.14^b^	124.35 ± 5.22^a^
5th	10	16.60 ± 0.30^b^	56.72 ± 0.75^b^	74.24 ± 0.75^ab^	84.82 ± 0.81^b^	107.91 ± 1.29^ab^	211.97 ± 2.87^a^	121.36 ± 4.78^a^

Means with different superscripts within the same column and class are statistically different

*NS* non significant, *BW* birth weight, *6MW* 6 months weight, *WW* weaning weight, *YW* yearling weight, *18MW* 18 months weight, *PrWDG* pre weaning (birth to weaning), *PoWDG* post weaning (weaning to 18 months)

* Significant at 0.05

** Significant at 0.01

*Sex* Sex of the calf had significant (p < 0.01) effect on all of the growth traits studied (Table 1). Males were consistently heavier (p < 0.01) than female calves in all significantly affected variables. Male calves at birth were 1.42 kg heavier than female calves. The effect of sex on BW of calves has also been reported by several workers (Giday 2001; Amsalu 2003; Jirenga et al. 2006; Getinet et al. 2009; Ndofor–Foleng et al. 2011; Mekonnen et al. 2011). Similarly, male calves were significantly heavier than females by 4.74 kg at 6 MW and 4.48 kg at weaning age. This trend of weights between sexes was also evident in post weaning growth traits (YW and 18 MW). The effect of sex on growth performance traits obtained in this study is in complete agreement with results reported by Getinet et al. (2009) for Ogaden cattle breed of Ethiopia and Ndofor-Foleng et al. (2011) for Gudali and Wakwa cattle breed in Cameroon. Males grow more rapidly and reach a greater mature weight while females have slower rate of growth and reach maturity at smaller size due to the effect of hormonal differences in their endocrinological and physiological functions, longer gestation length of male and to selection pressure that was more intense on males than female calves (Koger and Knox 2009). As for the growth rate of calves, males were 11.58 gm/day heavier than female calves at PrWDG and 11.63 gm/day at PoWDG. Also, male grew approximately 43.66 % (42.08 kg), 18.9 % (18.21 kg), 10 % (9.64 kg) and 27.44 % (26.44 kg) faster than their female counter parts from birth to 6-months, 6- to weaning age, weaning age to 12-months and 12- to 18-months, respectively. Their differences in growth rate decreased as the age increased from birth to yearling age thereafter increased and reach highest at yearling to 18-months age implying that sex effect are more pronounced with age approaching puberty. This calls for a need to conduct selection within the breed. The significant effect of sex on PrWDG and PoWDG obtained in this study is in agreement with other results (Mekonnen et al. 2011). However, Aynalem (2006) has not observed significant effect of sex on PrWDG and PoWDG.

*AEZ* AEZ effect was not significant (P > 0.05) for PoWDG. However, AEZ was found to have a significant (P < 0.01) effect on BW, 6 MW, WW, YW, 18 MW and PrWDG. Calves which were calved in lowland had consistently higher (p < 0.01) weights than calves calved in midland. In midland area, almost all livestock in the moist and sub-moist climate are owned by smallholder with a variable intensity of crop-livestock integration. The sub-humid and humid zone is characterized by cultivation of perennial crop–livestock and cereal–livestock systems. In this area, land devoted to livestock production (grazing lands and uncultivated arable land) is low. Grazing land make up a very small proportion of the total area. Human and livestock population pressure, expansion of cultivation, increased grazing burden, over-grazing, soil fertility problems and environmental degradation are some of the major features in midland contributing to the shrinkage of grazing land thereby reducing the condition and productivity of animals. Compared to those of the midland AEZ, availability of grazing land, feeding resources and management of land and animal are the better in lowland. Consequently, the significant AEZ effect on growth performance traits considered could be attributed to variations in climate, location, grazing land, stocking effect, variations in pastures, differences in herd management and herdsman skills, availability of most of the herds with the best performance (true phenotypic) across the AEZs and overall differences in animal management practice, nutrition and herdsman skills. The lowland has comparatively better nutritional environment than the midland thus facilitating better performance of the breed. Environmental effects influencing performance of tropical cattle have been reported under various production conditions. Ebangi et al. (2002) reported the significant difference observed in growth traits within two main ecological zones is due to location difference.

*Season of birth* All the growth performance traits studied in Sheko cattle were significantly (p < 0.01) affected by season of birth. Significant seasonal variations may be due mainly to variations in feed and fodder availability as well as disease incidence (Bell 2006) in different seasons. Calves born in dry season had higher birth weights compared to those born in both main rainy and short rainy seasons. The reason being that dams which calved in dry season would have better pastures during the previous season therefore results in weight increase and better body condition of the pregnant dams during calving. This result is in agreement with that of Giday (2001) and Melaku et al. (2011) who reported heavier birth weights for Fogera calves born in dry season. Other studies, however, (Aynalem 2006; Mekonnen et al. 2011; Ndofor-Foleng et al. 2011) reported a higher birth weights for cows which had calved during the main rainy season. Calves born during the short rains were heavier than those born in the main rains and dry season calves by 3.10 kg and 5.30 kg at 6 MW, and 4.57 kg and 2.49 kg at 18 MW. The heavier 6 MW found is probably because calf crops calved during the short rains reach 6-months of age during main rainy season where feed is abundant. The highest weight in short rains obtained in this study is in agreement with other reports (Mekonnen et al. 2011) at 6 MW. As for 18 MW, the reason for higher weight in short rainy season-born calves is due to favorable feeding conditions of 18 MW calves during the post weaning season. However, this result disagrees to those of Ebangi et al. (2002), Aynalem (2006), Szabo et al. (2006) and Ndofor-Foleng et al. (2011) who found heaviest 18 MW in those born during the dry season.

In case of dry season, calves born in dry season were 2.21 and 3.50 kg heavier at weaning age than short rainy and main rainy season-born calves, respectively which might be due to the fact that dams calved during the late dry season calves exposed to the earlier part of the short rainy season and the coming main rainy season, when the availability of feed resources (grasses and browses) is abundant for prolonged period of time. The combinations of short rainy and main rainy season very often result in excess forage in both quantity and quality leading to high milk production of cows for calf consumption. Similar, higher WW of calves born in dry season than other seasons has also been reported (Ndofor-Foleng et al. 2011) for Gudali calves in Cameroon. This finding disagrees with other workers (Mekonnen et al. 2011; Melaku et al. 2011) who reported higher WW of calves born during the short rains. On the other hand, calves born during the main rains were heavier than those born in the dry and short rainy season by 3.69 and 2.91 kg at YW, respectively. Heavier YW for calves born during main rains might be attributed to the variation in the amount and distribution of rain fall between season that has influence on availability of feed production and performance of cows. This result is in agreement to a number of workers, who reported heavier live weight at yearling (Ebangi et al. 2002; Szabo et al. 2006; Ndofor-Foleng et al. 2011) for calves born during the main rains. Other studies, however, reported heavier YW (Aynalem 2006) for calves born during dry season.

Season of calving had also a strong significant (P < 0.01) effect on PrWDG but not significant (p > 0.05) on PoWDG (Table 1). Lack of significant effect of season on PoWDG reported here is in agreement with other findings (Getinet et al. 2009; Melaku et al. 2011). Calves born during short rainy season had higher PrWDG than the other two seasons, which could be due to favorable feeding conditions of dams during this and the latter seasons, though had higher milk yield. This result is in agreement with that of Mekonnen et al. (2011) who reported higher PrWDG for calves born during short rains. Other study, however, reported high PrWDG calves born during dry season (Aynalem 2006). The significant effect of season of birth on growth performance of calves obtained in this study is in agreement with the results reported by Kassa-Mersha and Arnason (1986) in Ethiopia and Ndofor-Foleng et al. (2011) in Cameroon.

*Parity* Parity had a significant (at least P < 0.05) effect on all growth performance traits. The general trend in calves BW, 6 MW, 18 MW and PoWDG were increased as the parity increased from the first parity to the third parit*y* after which there was a declining trend to the fifth parity implied that these traits attain their maximum at the third parity. Similarly, WW and YW were increased as parity increased from the first parity to the fourth parity after which decreased in the fifth parity implied that these traits attain their maximum at the fourth parity. In this study, however, the trend of PrWDG was irregular and no specific trend was observed. Cows which calved for the first time had lightest (p < 0.01) calves at birth than the other higher parities whereas cows on their third parity had the heaviest BW. This variation could be attributed to competition for growth of the young dam and the fetus for the available nutrients, and dams as first-time calvers may produce less milk than average whereas mature cows provided a good maternal environment to the developing fetus. This is in agreement with research conducted by Mekonnen et al. (2011) who found lightest (p < 0.01) calves on birth weight of Horro calves at first parity. The significant (P < 0.01) effect of parity on birth weight of Sheko calves obtained in this study is in agreement with other reports (Giday 2001; Getinet et al. 2009; Mekonnen et al. 2011). However, some studies (Addisu et al. 2010; Melaku et al. 2011) reported non-significant effects (p > 0.05) of parity on birth weight. Likewise, calves of first parity were also lighter at 6, 9, 12 and 18 months of age than those of the other parities.

Regarding WW, calves of the fourth parity had the highest weaning weight, might be due to well developed mammary tissue of their mature status has contributed to reveal better maternal environment in terms of milk for the suckling calf. WW were significant between calves of the first, second and third parity. Significant effect of parity on weaning weight has also been reported by other workers (Getinet et al. 2009; Mekonnen et al. 2011; Melaku et al. 2011). As for YW, calves of the fourth parity had the highest YW. Yearling weight of calves from the fourth parity was insignificantly higher. Similar, significant (p < 0.01) effects of parity on YW were observed by Giday (2001) and Getinet et al. (2009). In case of 18-months calves’ weight, the third parity was significantly higher than the other parities except for the fourth parity. This result is similar to other findings (Getinet et al. 2009). Now a day, it is widely accepted that post weaning growth of calves is partly determined by the direct genetic effect of the calves and the level of environmental factors.

Parity had a significant effect on PrWDG (p < 0.05) and PoWDG (p < 0.01) of Sheko calves. The highest PrWDG was attained in 4th parity (224.94 ± 5.06 g/day) and the least in 5th parity (211 ± 7.44 gm/day). Whereas PoWDG was highest in 3rd parity (136.97 ± 2.90 gm/day) and the least in 5th parity (121.36 ± 4.78 gm/day). This might be due to the old age of the dams that leads to reduction in milk yield and that affects the birth weight and subsequent growth rate of the calf. PrWDG and PoWDG also took similar trend for parity effect except for the third and forth parity which showed a reversed trend. Significant effect of parity on PrWDG and PoWDG obtained in this study is in agreement with Melaku et al. (2011). However, this result disagrees to those of Addisu et al. (2010) who found non-significant (p > 0.05) effect of parity on PrWDG.

### Reproductive performance

Least squares means and standard errors for effects of non-genetic factors on reproduction traits of Sheko cattle breed is presented in Table 2. The coefficient of determination (R^2^) was 0.69 for DO and 0.70 for CI. The overall least squares means estimated for reproductive traits in this study were 248.32 ± 6.02 days for DO and 17.40 ± 0.20 months for CI. Generally, CI and DO of Sheko cows obtained in this study was longer to achieve recommended CI values of 365–385 days (Peters 1984). There is a need for management efforts to reduce the longer CI found in this study to achieve recommended CI that is associated with differences in management practices coupled with scarcity and deterioration of available feeds, poor breeding management, disease and parasites load and prolonged suckling by calves under extensive management systems.

**Table 2:** Least squares means (±SE) for effects of agro ecology, season and parity on days open and calving interval

Effects and levels	N	DO (days)	CI (months)
Overall	75	248.32 ± 6.02	17.40 ± 0.20
R^2^		0.69	0.70
C.V. %		13.16	6.24
AEZ		**	**
Lowland	43	274.63 ± 6.16	18.35 ± 0.20
Midland	32	220.53 ± 6.17	16.54 ± 0.20
Season		**	**
Dry	20	279.41 ± 14.53^a^	18.59 ± 0.48^a^
Short rainy	31	220.29 ± 7.35^b^	16.42 ± 0.24^b^
Main rainy	24	243.04 ± 10.96^ab^	17.32 ± 0.36^ab^
Parity		**	**
1st	22	289.02 ± 9.93^a^	18.79 ± 0.33^a^
2nd	14	250.29 ± 10.67^b^	17.65 ± 0.35^b^
3rd	12	267.75 ± 8.58^ab^	18.04 ± 0.28^ab^
4th	17	218.73 ± 14.25^cb^	16.66 ± 0.47^cbd^
5th	10	212.12 ± 15.54^cb^	16.08 ± 0.52^c^

Means with different superscripts within the same column and class are statistically different

*CI* calving interval, *DO* days open

* P < 0.05; ** P < 0.01

*AEZ* The effects of AEZs were significant (p < 0.01) for both CI and DO (Table 2). Cows which had calved in lowland were shorter (p < 0.01) CI and DO than cows which had calved in midland. As the breed is reared in different AEZs and having had different environments and production systems, the cows could vary in the level of performance. Cows from the lowland were consistently higher (p < 0.01) than midland cows in both DO and CI. Feeding resources, size and quality of grazing land, management of natural resources base including livestock and true phenotype of Sheko are becoming important factors influencing and determining the variation of the two AEZs. The midland AEZ are characterized by problem of water logging, problem of soil fertility, densely populated, shortage of grazing land (due to human and livestock population pressure) accompanied by feed shortage, increased grazing burden, subjected to overgrazing and environmental degradation, thereby limiting grazing space for the animals. Moreover, the size and quality of communal grazing land have been substantially reduced over the past 5 years. While in the lower altitude, grazing land, availability of true phenotypic (probably genetics) and management of animal and land were comparatively favourable for cattle production that brought shorter reproductive performance (CI and DO) of Sheko cows. Hence, more emphasis should be given to provision of satisfactory nutrition and good managerial effort to reduce DO and CI in order to improve the productivity of the breed. The significant effect of agro ecology on CI obtained in this study disagree with reports of Solomon et al. (2009) who reported non-significant effect of AEZ on CI for dairy cattle under smallholder management system in North-eastern Amhara Region, Ethiopia.

*Season* Season of calving had a significant (p < 0.01) effect on DO and CI. The significant effect of season of calving on reproductive traits could be attributed to the changes in climatic conditions and feeding regimes during different seasons. The cows that calved during the short rainy season had short DO and CI than those calved during dry and long rainy season (Table 2). They had 59.12 and 22.75 days DO and 2.17 and 0.90 months CI less than to those born in the dry and main rainy season. This is so because, the length of the rainy season is getting longer when short rainy-season is exposed to the following main rainy season. Thus when rainy season is prolonged or during this time feed availability is abundant and productivity of animals is generally high. In general, proper management and provision of satisfactory nutrition shorten CI and DO. The present finding is in agreement with the reports of Solomon et al. (2009) for Zebu cows, Melaku et al. (2011) for Fogera cows, Mekonnen et al. (2011) for Horro cows who reported a shorter calving interval for cows which had their calves during the short rainy season. Other studies conducted by Yifat et al. (2009) working on reproductive traits of CI and DO have reported a shorter values for cows which had their calves during the long rainy season. The significant effect of calving season on DO and CI were also reported by several authors (Giday 2001; Solomon et al. 2009; Melaku et al. 2011; Mekonen et al. 2011). However, non significant effect of calving season was reported by Aynalem (2006) and Getinet et al. (2009).

*Parity* CI and DO were significantly (p < 0.01) affected by parity. In contrast, Kefena (2006) reported non-significant effect of parity on CI and DO. The general declining trend was observed with the advance of parity number for the length of CI and DO after the first parity except for the 3rd parity which showed a slight but non-significance increase compared to the 2nd parity. This may calls for preferential treatments of cows for shorter CI and DO. According to Bulman and Wood (1980), the general declining trend of the length of CI and DO with the advance of parity and the incidence of silent estrus was highest in the primiparous cows and decreased with the advance in parity number and higher nutrient requirement for growth in addition to milk production and maintenance thus delays the onset of postpartum heat. The observed general trend of influence of cow parity on CI and DO is in general agreement with the results reported for indigenous and crossbred cows in Ethiopia (Negussie et al. 1998; Demeke et al. Demeke et al. 2004). The cows in their first parity had longest DO (289.02 ± 9.93) (at least P < 0.05) and CI (18.79 ± 0.33) (at least P < 0.05) whereas cows in fifth parity showed shortest DO (212.12 ± 15.54) and CI (16.08 ± 0.52) from the other parities. This result is in agreement to a number of workers, who reported the longest DO (Asheber 1992; Giday 2001) and CI (Giday 2001; Getinet et al. 2009) in young cows which might be due to lower energy balance as they are not able to consume more for their own growth, production, reproduction and maintenance, thus lower energy balance delays the onset of postpartum heat. The shortest calving interval in fifth parity might be due to their physiological maturity that contributed to have shorter CI and DO. Similar significant effect of parity on DO (Giday 2001; Getinet et al. 2009) and CI (Mureda and Zeleke 2007) has also been reported.

### Milk production performance

Least squares means and standard errors for effects of non-genetic factors on milk production traits are summarized in Table 3. The overall performance levels of Sheko cattle for DMY, 305DY, LMY and LL were 2.79 ± 0.06 lt, 839.07 ± 31.34 lt, 850.69 ± 24.16 lt and 307.69 ± 6.13 days, respectively. Performance levels [DMY, LMY, LL and stage of lactation (early lactation, mid lactation and late lactation)] of Sheko cattle reported in this study are higher than the previous report (Takele 2005) of on-farm survey for the same breed. These variations in performance levels are mainly due to difference in management practice that bring about differential response within the same breed and thus through good management such traits could be substantially improved. DAD-IS (2000) reported lower values of 7 month (210 days) for LL and 420 kg for LMY of Sheko cattle. Similarly, LL and DMY obtained in this study were higher than values reported by other studies for Fogera cattle (Addisu et al. 2010) and Barka cattle (Million and Tadelle 2003) but lower than N’Dama cattle in Gambia (Agyemang et al. 1991). On the other hand, the overall mean LMY (lt), Best 10 % (lt), and Best 25 % (lt) estimated were 850.69 lt, 1301.15 lt and 1139.49 lt, respectively, while the mean DMY, Best 10 %, and Best 25 % are 2.79 lt, 3.78 lt and 3.58 lt, respectively, were high indicating that improvements in productivity of animals on the basis of performance could be done with reasonable degree of accuracy. Milk yield improvement would require improving management practices or reducing environmental effects. Thus, the result obtained in the present study would indicate that both management and selection are important in improvement of the milk performance of the herd.

**Table 3:** Least squares means (±SE) for effects of agro ecology, season and parity on milk production traits

Effects and levels	N	DMY (lt)	N	LMY (lt)	N	305DY (lt)	N	LL (days)
Overall	1522	2.79 ± 0.06	1522	850.69 ± 24.16	547	839.07 ± 31.34	75	307.69 ± 6.13
R^2^		0.08		0.32		0.77		0.20
CV (%)		21.05		196.38		10.78		17.55
AEZ		NS		*		NS		*
Lowland	882	2.82 ± 0.10	882	855.85 ± 33.98	313	721.27 ± 33.75	43	310.26 ± 9.34
Midland	640	2.65 ± 0.11	640	743.89 ± 35.82	234	751.73 ± 27.85	32	283.73 ± 9.85
Season		NS		**		**		**
Dry	406	2.64 ± 0.17^a^	406	684.92 ± 56.80^a^	145	497.88 ± 52.55^a^	20	273.92 ± 15.62^a^
Short rainy	629	2.80 ± 0.08^a^	629	902.96 ± 28.77^b^	226	881.00 ± 23.55^b^	31	324.08 ± 7.91^b^
Main rainy	487	2.77 ± 0.13^a^	487	816.23 ± 44.76^ab^	176	830.61 ± 34.816^b^	24	292.99 ± 12.31^a^
Parity		NS		*		**		NS
1st	436	2.56 ± 0.13^a^	436	680.73 ± 42.75^a^	160	544.47 ± 56.45^a^	22	283.30 ± 11.75^a^
2nd	294	2.74 ± 0.15^a^	294	770.05 ± 52.19^ab^	102	771.53 ± 41.48^b^	14	284.75 ± 14.35^a^
3rd	243	2.88 ± 0.12^a^	243	866.96 ± 38.83^b^	87	846.06 ± 35.24^b^	12	297.92 ± 10.68^a^
4th	345	2.83 ± 0.21^a^	345	894.37 ± 69.76^b^	124	745.36 ± 55.00^cb^	17	320.41 ± 19.18^a^
5th	204	2.67 ± 0.19^a^	204	794.74 ± 64.11^ab^	74	772.05 ± 37.59^b^	10	298.60 ± 17.63^a^
Stage of lactation		**		NA		NA		NA
1st stage	450	3.88 ± 0.08^a^						
2nd stage	390	2.77 ± 0.07^b^						
3rd stage	682	1.8 ± 0.07^c^						

Least squares means with same superscript in the same column and class indicate non significance

*N* number of records, *DMY* daily milk yield, *LMY* lactation milk yield, *305DY* 305 days milk yield, *LL* lactation length, *NA* not applicable

* P < 0.05, ** P < 0.01

*AEZ* AEZ was significant (p < 0.05) source of variation for LMY and LL but not significant (p > 0.05) for DMY and 305DY. When the different AEZs were compared, cows in the lowland were higher (p < 0.05) than cows in midland in both LMY and LL. Obviously, as the breed is reared in two different agro ecological zones and having had different agro-climate, environment, production systems and grazing managements, the level of milk production performance varied accordingly. In lowland, the attention given in terms of feeding resource for animals is better than the midland. As the breed is well adapted in low-lying warm and humid environment (Takele 2005), these higher LL and LMY obtained in this area for Sheko cows in the present study compared to literature reports is partly due to high adaptation of this breed to live and produce in this agro-climatic and management condition and hence farmers in lowland tends to have more traditional knowledge in managing the breed. This knowledge provides a basis for local-level decision making in natural resource management, how to use an animal, food security and various other community-based activities for improved livestock production. While in midland, the emphasis given to improve livestock productivity and proper management of the natural resources base including grazing and livestock resources is relatively low. Moreover, expansion of cropping and land grabbing for cultivation is causing shrinkage of grazing lands and loss of key resources for livestock grazing thereby limiting feeds for animals which ultimately lower the production capacity of animals. As a result, the significant AEZ effect could be attributed to variations in grazing area, variation in adaptation to the natural environment and differences in traditional knowledge of animal management. Cattle in lowland were superior compared to those in midland. Therefore, in the smallholder midland, more attention should be given to improve livestock productivity and proper management of the grazing land. The significant effect of AEZs on LMY and LL obtained in this study is in agreement with other study (Solomon et al. 2009).

Season of birth: Season of calving was significant (p < 0.01) for LMY, 305DY and LL but not significant (p > 0.05) for DMY (Table 3). When LMY and 305DY studied, cows that calved in short rainy season had higher (p < 0.01) values than those cows which were calved during the dry season but the values were not statistically different (p > 0.05) with the rainy season calving whereas the lowest values was found for cows that calved in dry season. In case of LL cows that calved in dry and main rainy season were not statistically different (p > 0.05) whereas cows that calved in short rainy season had higher (p < 0.01) values than the previous two. Similar results to the present study were also reported by Giday (2001) and Melaku et al. (2011) studies with Fogera cows showed that short rainy season calving cows had higher LMY, 305DY and LL. The cows calving in short rainy season produced the higher LMY, 305DY and LL, mainly due to cows calved in this season further continued in the next rainy season characterized by excess succulence forages with high nutrient content, low fiber content, high digestibility and high voluntary intake by animals. Thus when nutritional adequacy is prolonged or during this season animals become productive for high milk yield performance. Whereas cows calving in dry season produced the lower LMY, 305DY and LL, may be due to low availability of natural forage and high temperatures during the dry season. These results indicate that calving in dry season and long rainy season is undesirable. Though breeding should be in a way that most calving occurs in short rainy season. To alleviate the problem of feed shortage during the dry season excess forage during the rainy season should be conserved in the form of hay. The significant effect of season on milk production traits of LMY, 305DY and LL observed for Sheko cows in this study is in agreement with other reports on crosses of local and Holstein–Friesian cows (Aynalem 2006; Amasaib et al. 2011) and Horro cows (Mekonnen et al. 2011).

*Parity* Parity effect was not significant (P > 0.05) for DMY and LL. However, parity was found to have a significant effect on LMY (P < 0.05) and 305DY (P < 0.01). Similarly, significant effect of parity on LMY and 305DY has also been reported (Aynalem 2006; Mekonnen et al. 2011). In contrast, Kefena (2006) reported a significant influence of parity on LL. Cows in their first parity had significantly lower LMY (P < 0.05) and 305DY (P < 0.01) than those cows in the higher parities due to the fact that first-lactation cows have lower energy balance for growth and lactation as they cannot consume adequate energy in the diet. However, LMY of first parity was not statistically different from that of 2nd and 5th parity. Whereas 305DY of first parity is statistically differ from all other parities. The result obtained in this study is in agreement with other workers who reported lower values of LMY and 3o5DY (Aynalem 2006; Mekonen et al. 2011) for cows in their first parity. It was observed that LMY was increased gradually as parity number increased from the first parity to fourth parity after which declined at the 5th parity implied LMY reached maximum at the fourth parity. Similarly, 305DY reached peak yield at the 3rd parity and subsequent fall implied that this trait attain their maximum at the third parity. Like other many studies the peaked yield of LMY and 305DY at fourth and third parity, respectively was mainly due to the increase in body weight combined with advancing age at full development of secretory tissues of the udder. The decreased LMY and 305DY observed after fourth and third party might be due to the physiological activities of all body systems start to decrease and the secretory tissues of mammary gland is partially degenerated leading to gradual decrease in milk production with advancing age. In other of the studies, however, Aynalem (2006) reported LMY peaked numerically at the second lactation. Similarly, the highest 305DY in third lactation is in agreement with that of Mekonnen et al. (2011) who reported peak 305DY at the third parity for Horro cows. The present study showed to determine the maximum parity where production decline that could enable to make proper culling. Although milk yield increased with increase in lactation length, yet it did not seem advantageous to have lactations exceeding 1 year. Moreover, longer lactations prolong the calving interval, thereby decreasing the number of calves that could be obtained during the life span of a cow. Therefore, attempts should be made to select cows on the regularity in breeding so that they should produce calves each year with a lactation period of about 10 months.

*Stage of lactation* The results revealed that stages of lactation was found to have a significant (p < 0.01) effect on DMY. The significant (P < 0.01) effect of stage of lactation on DMY has also been reported by other studies (Belay et al. 2012). However, Gadmade (1999) have reported non-significant effect of stages of lactation on DMY. It was observed that DMY significantly (p < 0.01) decreased as stages of lactation increased from early to late lactation. The highest milk yield was recorded in 1st stage of lactation and lowest was in 3rd stage of lactation. For example, there was a decrease of 2.08 litre/day of DMY from first stage to the third stage of lactation (Table 3). An early on-farm work reports on the same breed observed that daily milk yield significantly (P < 0.01) decreased with stages of lactation (Takele 2005) but the values reported were lower than the results of the present monitoring study. The comparatively higher values of stages of lactation for first, second and third recorded in the current study than previous reports of 3.3, 2.3 and 1.3 litres for the corresponding stage of lactation, with an overall average of 2.3 L per day might be attributed to the difference in the level of management. This is so because monitoring studies required to capture the full picture of the actual performance under the existing management system and resulted in higher stages of lactation. The difference might also be related to inter-annual natural effect. The result of present study agrees with different workers (Belay et al. 2012) who reported highest DMY in 1st stage of lactation and lowest DMY in 3rd stage of lactation.

## Conclusions

Results revealed that almost all non-genetic factors were observed with significant influence on growth, reproductive and milk production traits. Sex has an effect on all of the growth traits. Male are heavier and grew faster than female calves until post weaning growth period. AEZ has an effect at most live weight, in all reproductive traits and for two milk yield traits considered, indicating differences in agro–climate adaptation, production system, grazing area, management practice and availability of true phenotype of Sheko in the environments to which the breed is raised. Season of birth affected all of the reproductive traits, and many of the growth and milk production traits. Results suggest dry season calves give rise to heavier calves at weaning while short rainy season calves give rise to heavier calves at 6-, 18-month, and PrWDG. Whereas the main rainy season calves give rise to heavier calves at 12-month suggest seasonal differences in growth traits showed variation for different season. For milk yield traits, calves born in short rain season performs better LYD, 305DY and LL than others while for reproductive traits, calves born in dry season performs better DO and CI than others. The trend observed for most live weight and milk yield traits increased with increasing parity from the first to the third considered in the present study whereas the trend for reproductive rate (CI and DO) gradually decreased with increasing parity. Results of the study also revealed that growth, milk yield and reproductive traits considered for Sheko cows were found to be low. Feeding resources, management levels and true type Sheko cattle are important factors influencing and determining the productivity of the breed. It is apparent that most progress can be achieved through management factors and improving the level of nutrition. Results of the present study highlight the relevant factors affecting variability of traits considered and this give useful information for preferential treatments. Though, most of the factors exerted great influence, calls for further improvement of herd productivity. It was also observed that milk yield were higher at the early lactation stage, then it decreased linearly up to the end of late stage of lactation Generally, the effects of non-genetic factors on growth, reproductive and milk yield traits considered were significant and hence will need to be considered in cattle breed improvement program.
